# Tissue Specific Heterogeneity in Effector Immune Cell Response

**DOI:** 10.3389/fimmu.2013.00254

**Published:** 2013-08-27

**Authors:** Saba Tufail, Khan Farheen Badrealam, Asif Sherwani, Umesh D. Gupta, Mohammad Owais

**Affiliations:** ^1^Interdisciplinary Biotechnology Unit, Aligarh Muslim University, Aligarh, India; ^2^National JALMA Institute for Leprosy and OMD, Agra, India

**Keywords:** heterogeneity, effector T cell, homing, dendritic cells, chemokine receptors

## Abstract

Post pathogen invasion, migration of effector T-cell subsets to specific tissue locations is of prime importance for generation of robust immune response. Effector T cells are imprinted with distinct “homing codes” (adhesion molecules and chemokine receptors) during activation which regulate their targeted trafficking to specific tissues. Internal cues in the lymph node microenvironment along with external stimuli from food (vitamin A) and sunlight (vitamin D_3_) prime dendritic cells, imprinting them to play centre stage in the induction of tissue tropism in effector T cells. B cells as well, in a manner similar to effector T cells, exhibit tissue-tropic migration. In this review, we have focused on the factors regulating the generation and migration of effector T cells to various tissues along with giving an overview of tissue tropism in B cells.

## Introduction

**Figure 1:**
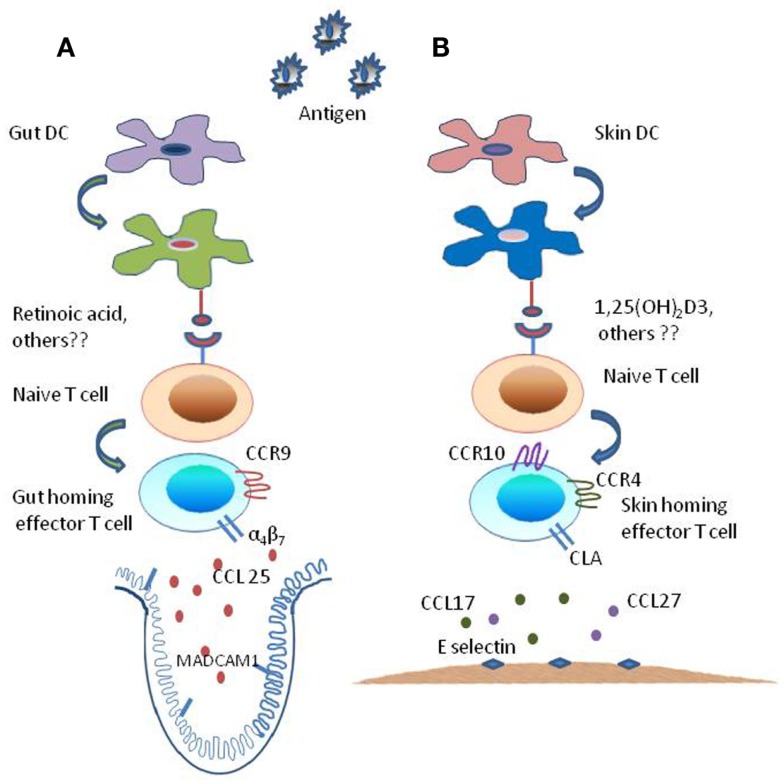
**Mechanism of generation of tissue specific effector T subsets**. The tissue-imprinting-receptors expressed on effector T cells are induced by signals derived from regionally imprinted dendritic cells (DCs); expression of α_4_β_7_-integrin and CLA are induced at low levels on T cells activated by DCs, this expression of imprinting receptor-repertoire is regulated by factors that are explicitly produced by gut- and skin-derived DCs. **(A)** Mesenteric lymph node or Peyer’s patch dendritic cells, i.e., intestine derived DCs along with external cues (Vitamin A metabolite retinoic acid) generate CCR9^+^ and α_4_β_7_^+^ gut-homing effector T cells by inducing α_4_β_7_-integrin expression and CC-chemokine receptor 9 (CCR9) expression on responding T cells while suppressing the expression of ligands for E-selectin. **(B)** By contrast skin-derived DCs along with external stimuli [Vitamin D_3_ metabolite 1,25(OH)_2_D3] generate factors that enhance the expression of ligands for E-selectin and CC-chemokine receptor (CCR10 and CCR4) while suppressing the expression of α_4_β_7_-integrin and CCR9 resulting in the generation of CLA^+^ and CCR4^+^ and CCR10^+^skin homing effector T cells.

Recent studies have revealed a diverse population of CD4 and CD8 T-cell effector subsets with distinct attributes in terms of phenotype, function, cytokine polarization, and anatomical distribution (1, 2). Homing of T-cell subsets to specific tissue sites is crucial for evoking a robust immune response combined with immunological memory (3). Activation of naive T cells in secondary lymphoid organs (SLOs) results in their differentiation to a heterogenous pool of effector T cells equipped to perform diverse functions (4). The heterogeneity of the effector T cells so generated partially owes to the site where progenitor naive T cells were initially activated (3). To this end, T cells activated in the lymph node of a particular organ or tissue acquire the “homing codes” and become destined to migrate accordingly in order to perform effector functions. It has been well documented that T cells primed in skin-draining lymph nodes display skin homing receptors whereas the intestinal lymph node provides the specific environment to the activating T cells to express mucosal homing receptors (Figure 1). Such decisive role in lymphocyte recirculation is tightly regulated by expression of particular adhesion molecules and receptors on lymphocytes, combined with the spatial and temporal expression of ligands for these receptors by a variety of tissue cells. The kind of tissue specific receptor-repertoire induced on the effector T cells is governed by the cues present in the lymph node microenvironment and external environment stimuli derived from food (vitamin A) and sunlight (vitamin D_3_) which themselves are influenced by tissue derived dendritic cells (DCs). In the present review, some fundamental concepts regulating the tissue specific heterogeneity in effector T cells has been discussed; emphasizing on cells, molecular mediators, and environmental signals that are responsible for imprinting specialized trafficking programs. Since tissue-tropic B-cell subsets have also been recognized, we have outlined briefly the mechanisms inducing tissue specific heterogeneity in B cells as well.

## Factors Influencing Tissue Tropism in Effector T Cells

### Molecular interactions

Studies dating back to 1970s had unveiled that adoptively transferred lymphocytes had a migratory pre-dilection for the tissues from which they were originally isolated (3, 5, 6). Lymphocytes isolated from mesenteric lymph nodes or Peyer’s patches (gut-associated lymphoid tissues) were found to populate preferentially mucosal effector sites (7–10). Later, “homing subsets” of T cells with distinct tissue tropism had been identified (11, 12). The selective expression of cellular adhesion receptors on T cells and vascular endothelium was found to be of prime importance in guiding T-cell subsets into and through distinct tissue compartments (13, 14). Since skin and intestinal tissues are the portal sites for pathogens entry, effector T cells infiltrate them enormously and this instigated the investigation of tissue-tropic homing of T cells at these sites. It has been evidenced that the α_4_β_7_-integrin and chemokine receptor CCR9 on T-cell surface target them to the lamina propria of the small intestine (3, 13–16). By contrast, skin homing T cells are characterized by expression of Endothelial cell selectin (E-selectin) and Platelet selectin (P-selectin) ligands in combination with CCR4 and/or CCR10 (3, 13–15, 17).

The homing receptors for intestinal tissues, α_4_β_7_-integrin and CCR9, interact with MADCAM-1 (mucosal vascular addressin cell-adhesion molecule 1) and CCL25 (CC-chemokine ligand 25 also called TECK) respectively expressed on endothelial cells of gut lamina propria venules (17, 18). α_4_β_7_-Integrin or MADCAM-1 blocking by antibodies and gene knock-out studies have indicated their role in trafficking CD4 and CD8 effector T cells to intestinal tissues (3, 15, 18–21). It has been found that the propensity of T cells to colonize intestinal mucosa is abrogated if the β_7_-integrin chain expression is hampered. Moreover, antibodies blocking either α_4_β_7_-integrin or MADCAM-1 could ablate inflammation in animal models of colitis and human subjects treated with antibody to the α_4_β_7_-integrin exhibited clinical and endoscopic remission of active ulcerative colitis (18, 22–25). α_4_β_7_-Integrin also seems to play a role in homing T cells to inflamed small intestine as blocking MADCAM-1 rendered T cells incapable of populating inflamed ileum in senescence accelerated mouse P1 (SAMP1)/yit mice (26). These studies overrule the plasticity of effector T cells with respect to α_4_β_7_-integrin in homing mucosal tissues during inflammation and make it seemingly feasible that α_4_β_7_-integrin “homing code” for intestinal mucosa is retained during mucosal inflammation as well. However, this hypothesis seemingly fails when the α_4_β_7_-integrin or MADCAM-1 antibody mediated ineffective treatment of ileitis induced by T-cell transfer from SAMP1 mice to severe combined immunodeficient (SCID) mice is considered (27). Likewise, β_7_-integrin deficient CD8 effector T cells were found to infiltrate small intestine and establish immunity to rotavirus infection (28). Hence, α_4_β_7_-integrin cannot be absolutely labeled a “guide” for T-cell homing to intestinal mucosa. The chemokine receptor, CCR9 is the other homing target for mucosal tissues. A blockade in CCL25, the ligand for CCR9, or genetic deletion of CCR9 itself severely limit the ability of effector T cells to localize to the small intestinal lamina propria and epithelium (15). T cells homing to large intestine (colon) although display α_4_β_7_ and α_4_β_1_ integrins but interestingly, in contrast to small intestine tropic T cells, a minority of T effector cells entering colon express CCR9 (15, 29). Moreover, unlike small intestine, colon does not express CCL25 and consequently CCR9 ablation or CCL25 blockade renders T-cell adhesion to colon unaffected. As such, T cells homing to colon are seemingly regulated by different mechanisms from those occurring in small intestine. In contrast to gastrointestinal tract, CCR9 does not act as the homing receptor for the effector T cells trafficking to liver or lungs (3). This indicates the selective tropism of CCR9^+^ T effector cells for the small intestine. However, CCR9 knock-out mice have been found to harbor normal small intestine tropic CD4 and CD8 T-cell pool and CD4 T effector cells lacking CCR9 have also been reported to colonize lamina propria of small intestine (30, 31). So, seemingly CCR9 is not absolutely required for T effector cell trafficking to small intestine.

Effector T cells with tropism for skin are characterized by the expression of cutaneous lymphocyte antigen (CLA) and chemokine receptors CCR4 and/or CCR10 (3, 15). CLA acts as a ligand for E-selectin as well as P-selectin and solely for E-selectin when expressed along with P-selectin glycoprotein ligand 1 (PSGL1) and CD43 respectively (32, 33). CLA interacts with E-selectin on skin endothelium and mediates tethering and rolling of T cells. CLA, CCR4, and CCR10 have been implicated in cutaneous T-cell homing under homeostatic as well as inflammatory conditions although entry of effector T cells in cutaneous tissues is limited during inflammation (3, 15). Augmented expression of E- and P-selectin has been observed during cutaneous inflammation and CD4 and CD8 effector T cells expressing counter ligands for the same to gain access to skin tissues (34, 35). Since E- and P-selectins are expressed on endothelium of many tissues during inflammation, T cells expressing their cognate ligands have been found to colonize those non-cutaneous tissues as well (36–38). So, it would be quite premature to call the E- and P-selectin ligands to be the exact “homing codes” in cutaneous effector T-cell trafficking. CLA^+^ CD4^+^ T cells express CCR4 and CCR10. CCL17 and CCL27, the ligands for CCR4 and CCR10 respectively, have been found to be displayed by both inflamed and non-inflamed skin endothelium (15). Although, generally the cutaneous expression of CCL17 is feeble, its expression is ameliorated during inflammation (39). Both CCR4 and CCR10 have been implicated in CD4 effector T-cell homing to delayed-type-hypersensitivity (DTH) induced inflamed skin (39, 40). However, the contribution of CCR4 and CCR10 to T-cell homing to inflamed cutaneous tissues depends on the kind of inflammation induced as only a small population of CD4 T cells have been found to express CCR10 in experimental *Candida*-extract induced and *Haemophilus ducreyi*-induced DTH and chancroid skin lesions respectively (3). In a manner similar to the above mentioned E- and P-selectin ligands, CCR4 and CCR10 cannot be absolutely made responsible for effector T-cell homing to cutaneous tissues. This is evident from the expression of CCR4 by a circulating human CLA^−^ T-cell subset and T-cell subsets in lungs and the synovial fluid of arthritis patients (3, 41). Reckoning with these observations, a cocktailed and orchestrated expression of homing receptors is seemingly required to control tissue specific targeting of effector T cells.

### Dendritic cells

#### Internal cues and dendritic cells

For antigen activated effector T cells to be “instructed” to home specific tissues like skin and intestines, cues are seemingly provided within the lymphoid tissues. T cells activated in skin-draining lymph nodes have been found to preferentially express CLA (42). Moreover, CD4 and CD8 T cells primed in skin-draining lymph nodes can bind E- and P-selectins while the T cells activated in mesenteric lymph nodes have been shown to exhibit an augmented expression of α_4_β_7_-integrin and CCR9 (43, 44). Although the expression of E-selectin ligands is seemingly specifically induced on CD4 and CD8 T cells activated in skin-draining lymph nodes, the expression of P-selectin ligand exhibits plasticity in this respect as it has also been shown to be expressed on CD4 T cells primed in mesenteric lymph nodes (3). These results demonstrate that T-cell priming in gut- and skin-draining lymph nodes renders induction of intestinal and cutaneous homing receptors respectively upon them.

Generation of gut-tropic and skin-tropic effector T cells in intestinal and skin-draining lymph nodes respectively is mediated by DCs. Stimulation of mouse CD4 and CD8 TCR-transgenic T cells with antigen pulsed-DCs isolated from intestinal lymph nodes induces expression of CCR9 and α_4_β_7_-integrin (3, 45, 46). Moreover, in the presence of these DCs, TCR-transgenic T cells with CD3 specific antibody exhibit expression of CCR9 and α_4_β_7_-integrin (3). DCs play a decisive role in the induction of CCR and α_4_β_7_-integrin expression since stimulation of CD8 T cells with peptide-pulsed mesenteric lymph node cells deficient in DCs could not induce expression of these homing receptors (47). α_4_-Integrin mRNA expression has been found to be increased in CD8 T cells primed with Peyer’s patch DCs which shows that α_4_β_7_-integrin expression is regulated at the level of α_4-_integrin expression but not β_7_-integrin expression (45). On the contrary, DCs from skin-draining lymph nodes induce increased expression of fucosyltransferase VII (Fuc-TVII, the enzyme involved in the synthesis of E- and P-selectin ligands and expression of CLA on human T cells), E- and P-selectin ligands, CCR4 mRNA as well as protein (48–50). However, plasticity appears in CCR4 expression as CCR4 has been found to be expressed on CD8 T cells activated in non-cutaneous lymph nodes *in vivo* (49). Thus, skin and intestinal lymph node specific DCs are capable of inducing tissue specific homing receptors on T cells both *in vitro* and *in vivo* leading to generation of tissue-tropic effector T-cell subsets.

#### External cues and dendritic cells

The internal cues in the lymph node microenvironment imprint tissue homing receptors on effector T cells but evolution has adapted two instances where external environmental stimuli “instruct” effector T cells for tissue specific homing (51–54). Vitamin A which exclusively enters the body through diet, has been adapted to induce small intestine homing properties (14, 51, 52, 55, 56) and vitamin D_3_ produced in skin on exposure to sunlight imprints T cells to achieve skin tropism (52, 57, 58).

Vitamin A (retinol) is converted to retinal which is further metabolized to retinoic acid by the catalytic action of retinal dehydrogenases (RALDHs) (3). Recent studies have demonstrated that induction of small intestine tropic homing receptors on responding T cells is a function of DC generation and presence of retinoic acid (52). Iwata et al. observed that addition of retinoic acid to activated mouse CD4 and CD8 T cells *in vitro* induces the expression of intestinal homing receptors α_4_β_7_-integrin and CCR9 while suppressing the expression of E- and P-selectin ligands (51). Importantly, the presence of RALDH inhibitor reduced the ability of mesenteric lymph nodes and Peyer’s patch DCs to generate gut-homing T cells and mRNA encoding RALDHs are expressed by DCs isolated from gut-associated lymphoid tissues (GALTs) but interestingly not by the DCs isolated from spleen. GALT-DCs perform better than splenic DCs in converting retinal to retinoic acid *in vitro* and they also upregulate α_4_β_7_-integrin and induce CCR9 on T cells better than the peripheral lymph node DCs (51, 52). Vitamin A metabolizing enzymes are also expressed by the intestinal epithelial cells and they may too impact gut T-cell responses but DCs being able to present both antigen and environmental cue retinoic acid to T cells seemingly are crucial in defining the efficiency and specificity of imprinting instructions received by T cells (52). Intestinal DCs can also carry antigen and gut-homing receptor cues to the draining mesenteric lymph nodes to present to naive T cells. Retinoic acid binds specifically to retinoic acid receptors (RARs) and the retinoid X receptors (RXR), the two families of nuclear receptors (3). An RAR antagonist blockade rendered mesenteric lymph node and Peyer’s patch DCs unable to induce α_4_β_7_-integrin on T cells (51). Moreover, in RAR response element reporter mice, RAR signaling is augmented on activation of CD8 T cells in mesenteric lymph node as compared to their activation in spleen (52). Together these results are suggestive that induction of gut-homing receptors on T cells is the result of selective ability of GALT-DCs to generate retinoic acid. However, peripheral lymph node DCs and splenic DCs induce expression of α_4_β_7_-integrin but not CCR9 on responding T cells *in vitro* (48). Splenic DCs have also been observed to be incapable of inducing α_4_β_7_-integrin on CD8 T cells in the presence of pan-RAR antagonist which indicates that splenic DCs may generate retinoic acid (3). CD103 DC subset, rapid inducer of RAR signaling upon T-cell activation, is specifically associated with induction of CCR9 (59, 60). Isolated splenic DCs which are incapable of inducing CCR9 *in vitro* also increase RAR signaling in responding CD8 T cells but not rapidly (52). Lamina propria (of the small intestine) populating CD103 DCs but not CD103 DCs of colon and lungs are potent inducers of CCR9 as well (59, 60). α_4_β_7_-Integrin mediates homing to both large and small intestine but CCR9 is more specifically required for small intestine homing (52). Thus, CCR9 is stringently regulated by retinoic acid. The preferential role of retinoic acid in small intestine is conceived since CD103 DCs from small intestine but not large intestine induce CCR9 (59). Moreover, non-intestinal DCs loaded with antigen when injected into lymph draining into mesenteric lymph nodes were found to induce the expression of CCR9 and α_4_β_7_-integrin on adoptively transferred TCR-transgenic T cells although not as efficiently as intestinal DCs (61–63). A recent study demonstrates that retinoic acid is necessary and sufficient for instructing DCs to regulate T-cell trafficking to gut (64). Moreover, the findings by Villablanca et al. indicate a crosstalk between the RAR and MyD88-dependant Toll like receptor signaling pathways. Retinoic acid seemingly induces the expression of α_4_β_7_-integrin and CCR9 on T cells by a direct effect of RA/RARα on Itga4 (α_4_-integrin expressing gene) and *Ccr9* gene promoters (14, 65–67). Recently, an essential role of retinoic acid has been dissected in the promotion of CD4 T-cell effector responses and RARα has been found to be a critical mediator of it (68).

Vitamin D_3_, formed by isomerization of previtamin D_3_ upon sunlight exposure in the skin, enters liver via circulation where it gets converted to 25(OH)D3 by the action of the enzymes 25-hydroxylases CYP27A1 or CYP27R1 and then in the kidney to the active form 1,25(OH)_2_D3 (69) by 1-hydroxylase CYP27B1 mediated catalysis which regulates calcium homeostasis by circulating systemically (52, 70–72). However, enzymes required for vitamin D_3_ metabolism have also been found to be expressed in keratinocytes (73), macrophages (74), and DCs (57). Both CYP27A1 and CYP27B1 enzymes are particularly present in human DCs which can therefore convert vitamin D_3_ to active 1,25(OH)_2_D3 (52). Thus, in a manner similar to vitamin A produced retinoic acid entering intestinal tract, vitamin D_3_ abundantly present within the skin itself are processed locally by DCs to active 1,25(OH)_2_D3 form. 1,25(OH)_2_D3 is an inducer of CCR10 expression on proliferating T cells (57). The addition of vitamin D_3_ in abundant concentration as produced in skin after sun exposure in the T-cell DC co-cultures induced the skin homing receptor CCR10 on responding T cells (52). CCR10 targets T cells from dermis to epidermis to bind to CCL27 secreted by the keratinocytes (75). Therefore, upon sun-exposure vitamin D_3_ may induce expression of CCR10 on activated T cells which are recruited to the dermis from systemic circulation. Since vitamin D_3_ is transported via systemic circulation, in some instances, CCR10 expression can be induced in skin-draining lymph nodes as well in addition to skin as a function of higher vitamin D_3_ concentrations. While the transport of intestinal retinoic acid to mesenteric lymph nodes is owed to gut DCs, if skin DCs can export vitamin D_3_ or its metabolites to peripheral lymph nodes remains elusive. 1,25(OH)_2_D3 inhibits rapid augmentation of α_4_β_7_-integrin on primed human T cells and reduces the ability of retinoic acid to upregulate α_4_β_7_-integrin and induce the expression of CCR9 (52). Of note, CLA and CCR4 (skin homing receptors) expression is slightly inhibited by vitamin D_3_ itself. 1,25(OH)_2_D3 was found to inhibit the expression of CLA specifically on effector CD4 cells which downregulated their migratory potential to the skin (76). These findings indicate that physiological regulation of skin T-cell phenotype and homing are quite complex as CLA and CCR4 are highly expressed by almost the complete pool of circulating CCR10 T cells *in vivo*.

1,24-(OH)_2_ D3 (tacalcitol), an analog of 1,25(OH)_2_D3, has also been found to downregulate the expression of CLA on effector CD4 T cells which prevented their skin infiltration, nevertheless, it could not render any effect on other homing receptors (77).

### Route of antigen administration, antigen dose, and presence or absence of adjuvant

Antigen administration route, antigen dose, and presence or absence of adjuvant are some of the other factors regulating tissue specific T-cell generation. Administration of ovalbumin orally in an ovalbumin-specific TCR-transgenic adoptive-transfer model resulted in robust expression of α_4_β_7_-integrin and CCR9 on responding T cells in mesenteric lymph nodes while antigen given intra-peritoneally induced expression of these receptors efficiently only when administered along with adjuvant (3). These may be because of differential targeting of DCs. Moreover, antigen dose has been found to influence RAR signaling induced gut-homing receptors (52). Low antigen dose pulsed-DCs can efficiently and specifically induce α_4_β_7_-integrin and CCR9 on responding T cells while DCs pulsed with high antigen dose could not induce α_4_β_7_-integrin and CCR9 efficiently on priming T cells thus reducing their gut-homing property (78). Upregulated selectin ligand expression related to skin homing in mouse is associated with high antigen doses. Whether high dose antigen induces generation of T cells migrating with lesser specificity (i.e., of more promiscuity) to other tissues *in vivo* remains obscure.

### Cytokines

Cytokines being crucial effectors and regulators of immune responses also seemingly regulate T-cell homing but is poorly understood. CLA expression on *in vitro* primed T cells is induced by the Th1 cytokine interleukin (IL)-12 whereas suppressed by the Th2 cytokine IL-4 (42, 52, 79). Interestingly, these cytokines exhibit opposite effect on the skin homing chemokine receptor CCR4 which gets upregulated by IL-4 and suppressed by IL-12 (80). Indeed, these conflicting regulatory events *in vitro* are not reflective of physiological expression patterns since CLA and CCR4 together direct the homing of T cells into the skin. It seems quite possible that *in vivo* homing specificity to skin requires a cocktailed and coordinated expression of specific receptor combinations along with coordinated activity of numerous cytokines. Also, *Aldh1a2* transcription is found to be diminished in IL-4rα^−/−^ mesenteric lymph node DCs which efficiently induce CCR9 on responding CD4^+^ T cells, indicating that IL-4 has a potential role to play in imprinting CD103 mesenteric lymph node DCs with gut-homing receptors (63, 81). Moreover, IL-15 has been found to modulate the effect of retinoic acid by promoting inflammation rather than oral tolerance to dietary antigens (82, 83). Therefore, physiological imprinting possibly is a function of combinatorial signaling together with sequential exposure to external environmental and cytokine cues during the evolution of antigen-dependent T-cell and DC responses.

## Tissue-Tropic Effector T-Cell Subsets in Internal Organs

Effector T cells have been found to traffic actively in some instances to non-inflamed extralymphoid tissues or internal organs (other than skin and intestine) as well (3, 84–86). For instance, intercellular adhesion molecule 1 (ICAM 1) and vascular cell-adhesion molecule 1 (VCAM1) mediate CD8 T-cell effector localization to non-inflamed liver (87). Moreover, CD8 effector T-cell homing to non-inflamed lung parenchyma is mediated by lymphocyte function-associated antigen 1 (LFA-1; CD11a–CD18) and is sensitive to pertussis-toxin which partially owes constitutive CCL5 expression in lungs (88). Also, CD4 T-cell blasts exhibit sensitivity to pertussis-toxin and dependence for LFA-1 and α_4_-integrin-VCAM1 while homing to un-inflamed spinal-cord parenchyma (89, 90). CD8 effector T cells primed with a model antigen (selectively expressed by epithelial tissues of the small intestine) in the gut draining lymph nodes home to a number of extralymphoid tissues like liver, kidneys, brain, and lungs (85). These findings elucidate that effector T cells trafficking these organs bypass the requirement of priming in draining lymph nodes of corresponding organs. On a similar line, post 3 days of intraperitoneal administration of antigen, intestinal lymph node derived CD8 effector T cells expressing CCR9 and α_4_β_7_-integrin are found lodging in the lungs and liver (47). Of note, it has been observed that T cells primed in the cervical lymph node, show preferential tropism for central nervous system (CNS) (3). Priming of mouse CD8 T cells with intra-cerebrally injected tumor cells in cervical lymph nodes induced expression of partially redundant but different adhesion molecules in comparison to T cells activated in mesenteric and inguinal lymph nodes. An upregulated expression of α_4_β_7_-integrin and P-selectin ligand was induced on T cells (44). Moreover, cervical lymph node primed effector T cells exhibit better access to CNS after being transferred to brain-tumor-bearing mice than their inguinal lymph node derived counterparts. Importantly, human subjects manifested with relapsing multiple sclerosis when treated with anti-α_4_β_7_-integrin chain humanized antibody (natalizumab) show health betterment highlighting a role of integrin in mediating localization of encephalitogenic T cells in CNS (91, 92). These findings indicate that T cells activated in the relevant draining lymph nodes of extralymphoid tissues (other than skin and intestine) may exhibit migratory pre-dilection for those tissues and imprinting of specific homing codes on T cells in draining lymph nodes may be harboring obscured complexity/may be a more generalized phenomena.

## Tissue Tropism in B Cells

In a manner similar to effector T cells, heterogeneity in the B-cell effector pool is also observed since plasma cells embody the attribute of homing to specific tissues. The development of effector B cells, i.e., antibody secreting cells (ASCs) or plasma cells is a multi factorial event and the underlying mechanisms that regulate the development of specific plasma cell have been reviewed (93). Generally, the site of antigen presentation and ASC differentiation (together with the nature of the stimulating antigen) determines the main immunoglobulin isotype that is expressed by the induced ASCs; thereafter, these ASCs can differentiate into sessile plasma cells that reside in the secondary lymphoid tissues of origin (a phenomenon particularly common for IgM ASCs) or traffic through the efferent lymph to the blood to populate distant sites for targeting the preferential production of specific antibody to mucosal surfaces, to lymphoid tissues, and to sites of inflammation. Rummage around the literature to dissect the mechanism for the differential homing of ASCs gave us the link that both the site of induction and the isotype expressed correlate with the homing potential and final tissue distribution of the resulting ASCs (7). Moreover, the existence of tissue tropism are regulated by chemokines and adhesion molecules which work together with environmental cues to mediated the distinct tissue trafficking patterns of various ASCs as in case of effector T cells.

It is found that the IgG ASCs populate the inflammatory sites whereas IgA-ASCs have homing specificity for mucosal tissues and both types of ASCs populate the bone marrow (8, 94, 95). It has been observed that a large fraction of IgG ASCs express L-selectin and α_4_β_1_ and upregulate their expression of CXCR3 (96–98). The ligand for α_4_β_1_ is vascular cell-adhesion molecule (VCAM1) and for CXCR3 are monokine-induced by interferon-γ (MIG/CXCL9) and (IP10/CXCL10), these ligands are expressed by inflamed tissues resulting in recruitment of the plasmablasts expressing α_4_β_1_ and CXCR3 to these sites. Thus, IgG ASCs probably using these receptor-repertoires traffic to inflamed tissues. Likewise, a large population of IgA-ASCs induced in SLOs express α_4_β_7_-integrin and CCR9 and CCR10 receptor and traffic to mucosal tissues which constitutively and differentially express α_4_β_7_ ligand MADCAM-1 and both chemokine ligands respectively (7, 16). It is to be noted that CCR10, a skin homing receptor for T cells is a mucosal homing receptor for IgA-ASCs; on the contrary, CCR9 remains the small intestinal traffic code for both the effectors, T cell and B cell. A significant role of the chemokine CCR10 and CCR9 in providing mucosal and small bowel immunity has been documented (7). Most IgA-ASCs in the lamina propria of the small intestine express CCR9 whereas the expression of CCR9 by IgA-ASCs from other segments of the gut is rare (99). In concordance with these observation the CCR9 ligand CCL25 are restricted to crypt epithelial cells and endothelial cells in the small intestine whereas the CCR10 ligand CCL28/MEC is expressed by most mucosal epithelial cells (for example, the large intestine, stomach, trachea and bronchi, mammary glands and salivary glands) and its cognate receptor is also expressed on all ASCs homing to mucosal tissues (99–102). However, it is more intriguing that even IgA-ASCs in the small intestine which in essence are responsiveness to CCL25/TECK (a ligand for CCR9) also express CCR10 (99); justifying the broad role of CCR10 chemokines in provoking immune response to antigens at mucosal tissues. The scenario gets more complicated, as in one instance IgA-ASCs are seen to require both CCR9 and CCR10 for efficient localization to small intestine (99), on the contrary, in another case redundant roles of CCR9 and CCR10 are showed for successful migration of IgG-ASC to small intestine (103). The reason for these incongruities remains to be elucidated, although a possible explanation may be inflammation induced upregulation of CCL28 in the small intestine which may suppress the dependence of ASC homing on CCR.

Bone marrow, the chief site for serum antibody production, harbors a large number of IgG and IgM ASCs and a smaller pool of IgA-ASCs. Systemically induced and intestinally induced IgG ASCs and IgA-ASCs respectively populate the bone marrow (95, 97, 104). The ability of IgG ASCs to migrate to the bone marrow is associated with their expression of CXCR4 and their responsiveness to CXCL12 expressed by bone marrow stromal cells. Because IgA-ASCs in mucosal lymphoid tissues maintain responsiveness to CXCL12, CXCR4 can probably mediate trafficking of IgA-ASCs to the bone marrow. Other chemokines responsiveness may contribute in the homing of ASCs to the bone marrow. CXCL16, a ligand expressed by bone marrow has its cognate receptor (CXCR6) expressed upon many ASCs populating bone marrow and numerous circulating CD38^+^ ASCs (of undetermined isotype) (105). Moreover, CCL28 may also have some role in the recruitment of CCR10-expressing cells (particularly IgA-ASCs) to the bone marrow and more importantly the ligands including α_4_β_1_, LFA-1, P-selectin ligands, and CD22 participate in ASC trafficking and/or survival in this site (95, 106, 107).

As discussed above, that plasmablasts or early ASCs leave the lymph nodes and Peyer’s patches to migrate to tissues via systemic circulation. During this migration they reduce responsiveness to chemokines expressed by the lymphoid tissues and gain responsiveness to chemokines expressed by the non-lymphoid tissues. In this regards, antigen-specific IgG ASCs generated in the spleen downregulate their responsiveness to CXCL13, CCL21, and CCL19 (chemoattractant for lymphoid tissues) although they retain responsiveness to CXCL12 (chemoattractant for bone marrow) (95). Moreover, ASC differentiation *in vitro* as well as *in vivo* renders deficits in expression of CCR6 (and responsiveness to CCR6). This loss of responsiveness (and/or receptor expression) to lymphoid tissue chemokines and parallel upregulation of expression of cognate receptors for tissue or inflammation selective chemokines facilitates plasmablast trafficking to specific tissues. Homologous chemokines CCL28 and CCL25 belonging to the chemokine subfamily differentially expressed by epithelial cells, attract IgG ASCs to the epithelial surfaces. CCL27 (keratinocyte-expressed chemokine), second ligand for CCR10 and CCL20, the CCR6 ligand are the other members of the same subfamily and these chemokines have seemingly evolved to coordinate immunity at exposed epithelial surfaces. A feeble expression of CCR10 is seen in upto one-sixth population of circulating IgG ASCs which may be responsible for lodging these cells to mucosal epithelial tissues particularly upper respiratory tract. Moreover, low levels of CCR10 expression combined with feeble expression of α_4_β_7_-integrin explain the low frequency of IgG ASCs observed in intestinal mucosal tissues. Also, upregulated expression of CXCR3 (and CLA) together with downregulated expression of CCR10 by IgG ASCs could facilitate their recruitment to the skin (where the other CCR10 ligand CCL27 is expressed) during chronic inflammation. From the above discussion it is clear that the differential interactions of ASCs with cell-adhesion molecules VCAM1 versus MADCAM-1 and their responsiveness to a subset of chemokines are likely to be an important for selective trafficking of IgA versus IgG ASCs, however, until today there is no clear data on the expression of other tissue specific adhesion molecules that might also contribute to the distribution of IgG or IgA-ASCs; moreover, the identification of newer chemokines and their cognate receptors along with cell-adhesion molecules may offers answers to our unsatisfied queries of differential homing of specific ASCs to specific sites.

As already discussed that DCs are essential for efficient T-cell activation, on the same line, DCs seemingly also influence B-cell responses by enhancing their differentiation to ASCs and survival. DCs have been shown to present unprocessed antigens to B cells *in vivo* and influence B-cell function in a tissue specific manner. For example, Peyer’s patch DCs have been found to induce class switching to IgA by activated B cells. Moreover, recently Mora et al. demonstrated that GALT-DCs but interestingly not DCs of other lymphoid organs induce gut-homing properties on primed B cells as they are essential for the coordinate expression of CCR9 and the IgA immunoglobulin isotype during the GALT response to small intestinal antigens (13); as induction of homing properties upon effector T cells is influenced by external cues like vitamins, similarly vitamin A has been found to imprint plasmablast homing to the small intestine (108, 109). As for T cells, retinoic acid presented by GALT-DCs during B-cell stimulation enhances α_4_β_7_ and induces CCR9 on the responding cells. DCs from mesenteric lymph node also enhance IgA production, an effect that is amplified by retinoic acid but also requires DC-expressed IL-6 or IL-5 (13). In contrast to the overlapping role of retinoic acid in imprinting small intestine homing properties on T cells and plasmablasts by inducing CCR9 expression, the signals inducing CCR10 expression on IgA plasmablasts are poorly understood. Moreover, the presence of high levels of RA in intestine and GALT promote B-cell class switching to IgA and thus boost the production of IgA in the intestinal mucosa (110). It is documented that RA can potentially interact with mechanisms inducing IgA-ASCs such as TGFβ, iNOS/NO, and probably others. However, the overall relevance of RA for TD and/or TI IgA responses *in vivo* as of yet remains to be unveiled. Of note, it is intriguing that gut-associated DCs and RA modulate intestinal immune responses by affecting both lymphocyte migration and effector activity (64).

Although 1,25(OH)_2_D3 mediated induction of CCR10 on human B cells is elucidated, but if this molecule has any part to play in inducing CCR10 on mucosal lymphoid tissue is yet to be determined. Deficiency of vitamin A (but not vitamin D_3_) has been correlated to shrinkage of IgA ASC pool in mucosa; induction of CCR10 expression on IgA plasmablasts does not require vitamin D or its receptor in mice and it is yet not clear whether the concentration of 1,25(OH)_2_D3 in mucosa associated lymphoid tissues is sufficient to induce CCR10 expression. Hence, CCR10 expression by IgA-ASCs are seemingly controlled by diverse mechanisms which are however independent of RAR or VDR signaling. Of note, in addition to DCs, macrophages possibly too play some role in B-cell homing to specific tissues since macrophages populating intestinal lamina propria have been observed to secrete retinoic acid which could be sufficient to induce gut-homing receptors on activated B cells but require further confirmation (110, 111). Thus by directing plasma-cell homing, these players might help to determine the phenotype and efficiency of mucosal, inflammatory, and systemic antibody responses.

## Conclusion

The heterogeneity in the effector T-cell population has become quite apparent and trafficking programs involved have been dissected to some extent with the identification of various adhesion molecules, chemokines together with the elucidation of the crucial role played by internal, external tissue environment, and DCs. Nevertheless, in spite of considerable advances made in understanding lymphocyte trafficking, numerous aspects of the imprinting and regulation of homing programs remain to be addressed. It is necessary to clarify whether generation of tissue specific effector T cells is limited to skin and intestine or is a generalized attribute of all tissue-draining lymph nodes. Moreover, cells, factors, and molecular mechanisms targeting lymphocyte migration to tissues other than skin and intestine remain yet to be unraveled. It is also of utmost importance to dissect the specific site where DCs are imprinted and the molecular mechanism behind this process together with elaborative conception of the molecular signals produced by DCs to induce tissue tropism in responding lymphocytes. Understanding the reason and the mechanism of differential expression of homing receptors on T and B cells targeting the same tissue is also important. Deciphering these unveiled issues will open avenues to novel prospects for therapeutic regulation of lymphocyte migration, with potential use in increasing the efficiency of vaccine-induced immunity and in restraining the pathologies associated with autoimmune and inflammatory diseases.

## Conflict of Interest Statement

The authors declare that the research was conducted in the absence of any commercial or financial relationships that could be construed as a potential conflict of interest.
